# Bidirectional association between symptomatic knee arthritis and circadian syndrome among middle-aged and older population: an analysis from the China health and retirement longitudinal study

**DOI:** 10.1186/s12889-025-25100-1

**Published:** 2025-11-03

**Authors:** Yasi Yang, Boran Sun, Wenbo Xiao, Aerman Nuer, Lemeng Ma, Yun Zhu, Yongjie Chen, Xiaochen Huai, Yuan Wang, Wenli Lu

**Affiliations:** 1 Department of Epidemiology and Statistics, School of Public Health, Tianjin Medical University, Tianjin, People’s Republic of China; 2 Tianjin Key Laboratory of Environment, Nutrition, and Public Health, Tianjin Medical University, 22 Qixiangtai Road, Tianjin, 300070 China; 3 Linking Innovations Co. Ltd, Beijing, China

**Keywords:** Chinese adults, Circadian syndrome, Symptomatic knee arthritis, Aging

## Abstract

**Background:**

Increasing evidence has shown that there was an association between arthritis and metabolism and circadian rhythms. Circadian syndrome (CircS) can serve as an overarching indicator of circadian and metabolic disturbances. However, there are limited studies investigating the longitudinal association between the symptomatic knee arthritis and circadian syndrome (CircS).

**Methods:**

This study used the data from two waves (2011 and 2015) of the China Health and Retirement Longitudinal Study (CHARLS). CircS was diagnosed when participants exhibited four or more of the following specified components: short sleep, depression, abdominal obesity, hyperglycemia, hypertension, and dyslipidemia. Symptomatic knee arthritis was diagnosed when both concurrent pain in knee joint and physician-diagnosed arthritis existed. Individuals diagnosed with CircS in 2011 were excluded from the symptomatic knee arthritis group, and those with symptomatic knee arthritis in 2011 were excluded from the CircS group. Each group was followed up for four years to observe the incidence of CircS and symptomatic knee arthritis, respectively. Binary logistic regression models were used to estimate the odds ratios (ORs) and 95% confidence intervals (*CI*s) for the longitudinal association between the symptomatic knee arthritis and CircS. Cross-lagged path analysis model was further conducted to estimate the bidirectional relationship. All models were considered to adjust for potential confounders.

**Results:**

At the baseline, 5525 non-CircS individuals and 9773 individuals without symptomatic knee arthritis were included in this study, respectively. During 4-year follow-up, 1278 CircS and 950 symptomatic knee arthritis new cases were observed respectively. After adjusting for potential confounders, symptomatic knee arthritis was associated with increased risk of CircS (OR = 1.58, 95% *CI*: 1.24–2.02). CircS was associated with increased risk of symptomatic knee arthritis (OR = 1.52, 95% *CI*: 1.31–1.76). Cross-lagged path analysis showed that the symptomatic knee arthritis significantly affected the incidence of CircS (*β*_1_ = 0.05, 95% *CI*: 0.03–0.07, *P* < 0.001), and vice versa (*β*_2_ = 0.07, 95% *CI*: 0.05–0.09, *P* < 0.001).

**Conclusions:**

Significant bidirectional associations were identified between the symptomatic knee arthritis and CircS. Interventions should be developed to prevent the development or progression of both symptomatic knee arthritis and CircS.

**Supplementary Information:**

The online version contains supplementary material available at 10.1186/s12889-025-25100-1.

## Introduction

Arthritis resulted in impaired quality of life and great economic burdens [1, 2]. Rheumatoid arthritis (RA) and osteoarthritis (OA) are prevalent, debilitating joint disorders [3, 4]. In 2020, approximately 595 million people worldwide were affected by osteoarthritis, while rheumatoid arthritis impacted around 17.6 million individuals [5, 6]. OA has been a leading cause of chronic pain and long-term disability [5]. Patients with OA are characterized by the chronic pains of joints, with the knee being the most commonly affected joint [3, 5]. The prevalence of symptomatic knee osteoarthritis (KOA) was 8.1% in China [7]. Consequently, there exists an immediate imperative to identify targeted risk factors for arthritis to reduce arthritis incidence.

The circadian syndrome (CircS) is defined by a cluster of risk factors, including obesity, hypertension, dyslipidemia, type 2 diabetes, depression, and sleep disorder [8]. Previous research illustrated that there was a positive association between metabolic syndrome (MetS) and OA [9]. Some results indicated that knee OA and hand OA, rather than OA occurred in other joints, was significantly associated with higher MetS risk [10] and individuals with MetS had progression of osteophytes of knee [11]. Additionally, previous surveys highlighted the contributions of both short sleep duration and depression to the elevated prevalence of arthritis and these factors were also associated with knee joint symptoms [12–15]. However, the traditional approach has typically involved analyzing these factors in isolation, without considering them collectively. The introduction of the CircS concept presents a viable and scientifically robust explanatory framework. Prior research has showed that CircS serves as a more reliable predictor for cardiovascular disease compared to MetS in China [8]. In the past few years, increasing evidence has shown that circadian rhythms have a strong effect on metabolism [16, 17] and there has been a strong interaction between arthritis and metabolism [18, 19], providing additional support for the hypothesis that CircS may have an interaction with symptomatic knee arthritis. However, there are limited studies investigating the association between the symptomatic knee arthritis and CircS to prevent the progression of patients. Thus, this study aimed to examine whether this bidirectional relationship exists using data from China Health and Retirement Longitudinal Study (CHARLS).

## Patients and methods

### Study population

CHARLS is a nationally prospective survey of middle-aged and older adults in China and informed consent was obtained from all participants. Additionally, the study protocol received approval from the Biomedical Ethics Review Committee of Peking University (IRB00001052-11015) and a detailed exposition of its introduction has been previously reported in the literature [20].

**Fig. 1:**
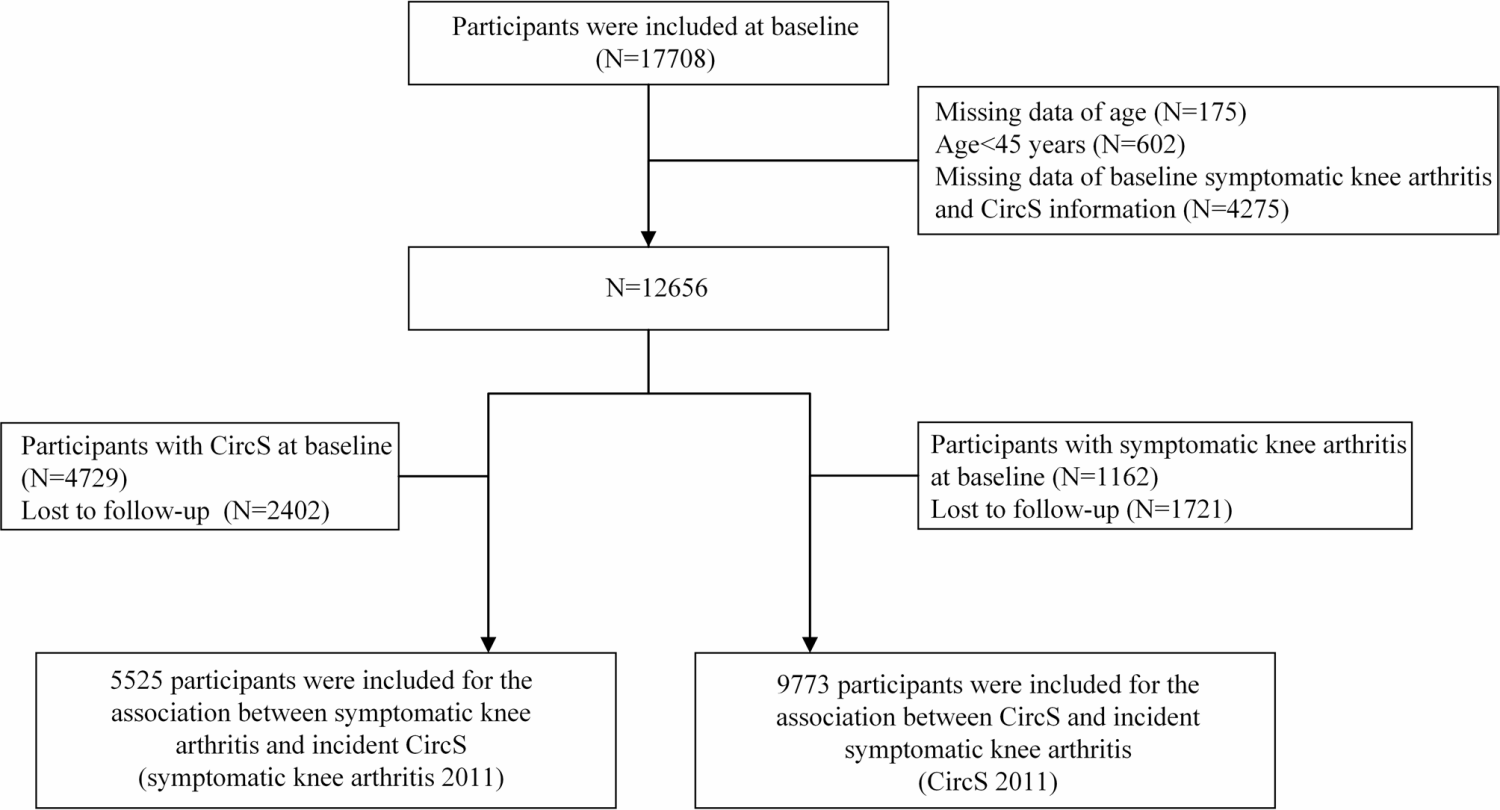
Flowchart on the sample selection

In this study, we used the 2011 and 2015 wave data in the CHARLS as the baseline and follow-up. At baseline, a total of 17,708 participants completed the baseline survey. The exclusion of participants was based on the following criteria: (1) missing data of age and age < 45 years, (2) missing data of baseline symptomatic knee arthritis or CircS information, (3) participants with prevalent CircS or symptomatic knee arthritis at baseline, respectively, (4) lost to follow-up. According to different exposures, 5525 participants and 9773 participants were included, respectively. The detailed flow chart of the study population selection is shown in Fig. 1.

### Definition of CircS

The diagnosis of CircS was based on the following components: (1) waist circumference of ≥ 80 cm for women and ≥ 85 cm for men; (2) elevated levels of triglycerides (≥ 150 mg/dL) or use of medication for dyslipidemia or ever had dyslipidemia; (3) low levels of high-density lipoprotein-cholesterol (< 40 mg/dL in men and < 50 mg/dL in women) or use of medication for the treatment of dyslipidemia or ever had dyslipidemia; (4) systolic blood pressure ≥ 130 or diastolic blood pressure ≥ 85 mmHg or use of medication for hypertension or ever had hypertension; (5) elevated fasting glucose (≥ 100 mg/dL) or use of medication for diabetes or ever had diabetes; (6) short sleep (< 6 h/day); (7) depression criteria (10-item version of the Center for Epidemiological Studies Depression Scale (CES-D −10) score ≥ 10). Participants with 4 or more of these risk factors were defined as CircS.

### Assessment of symptomatic knee arthritis

Consistent with the definition employed in the previous study [21], symptomatic knee arthritis was defined as simultaneous presence of physician-diagnosed arthritis and knee joint pain.

### Covariates

The covariates of interest included sociodemographic factors, lifestyle factors, and health status indicators. Sociodemographic characteristics included age, sex, education (elementary school and below, secondary school and above), marital status (married or partnered, and single or divorced or widowed or never married), residence (rural or urban), and annual household income (≤ 2000, 2001–10000, 10001–25000, >25000). Lifestyle characteristics included smoking, drinking, and moderate to vigorous physical activity (MVPA). Drinking was categorized as drinker or non-drinker. Smoking was classified as non-smoker, ex-smoker or current smoker. MVPA was defined as engaging in moderate or vigorous physical activity for at least 10 min in a usual week. Health status consisted body mass index (BMI), cancer, stroke and heart problem. According to the World Health Organization (WHO) definitions, BMI was divided into underweight (< 18.5), normal (18.5–24.9), overweight (25.0–30.0), and obesity (>30.0) categories [22].

### Statistical analysis

Continuous and categorical variables were presented as frequencies (percentages) and means (SD), respectively. Two-sample *t* test and Chi-square test were performed to compare baseline characteristics among groups.

To examine the association between the symptomatic knee arthritis and CircS, binary logistic regression models were utilized to calculate the odds ratios (ORs) and 95% confidence intervals (*CI*s) for the association. In addition, subgroup analyses were also conducted based on age (< 60 and ≥ 60) and sex.

Data of physical activity were collected in a random subgroup of almost two-fifths of all CHARLS participants. Therefore, it is reasonable to assume that the data is missing at random. Paul and his colleagues found that using multiple imputation improved efficiency of effect estimates and reduced bias at any proportion of missing data [23]. In this study, 5-fold multiple imputation was firstly performed to impute the missing values of MVPA. Binary logistic regression and subgroup analyses were then performed using the imputed dataset as the main results.

We employed a cross-lagged path analysis model, which is a widely used form of path analysis, to further explore the bidirectional relationship between the symptomatic knee arthritis and CircS using the imputed dataset. We also reconstructed the model after grouping by sex to explore whether sex had an impact on the bidirectional association. The study utilized a simplified model diagram (Figure S1 supplementary material). The path coefficient *β*_1_ represents the impact of symptomatic knee arthritis at baseline on CircS at follow-up, while *β*_2_ signifies the influence of CircS at baseline on symptomatic knee arthritis at follow-up. If *β*_1_ = *β*_2_, it indicates that symptomatic knee arthritis and CircS are reciprocally influenced in a bidirectional causal sequence. *β*_1_ > *β*_2_ suggests a primary effect from symptomatic knee arthritis to CircS, and vice versa. The difference between *β*_1_ and *β*_2_ was assessed using a Z-test. Model fit was evaluated using the Root Mean Square Error of Approximation (RMSEA) and Comparative Fit Index (CFI).

All models were adjusted for potential confounders, encompassing sociodemographic factors, lifestyle factors, and health status indicators. A comprehensive list of these variables, along with their detailed definitions, was provided in the covariates section. To confirm the robustness of our findings, sensitivity analysis was carried out with repeated binary logistic regression using the raw dataset without imputation. R (Version 4.3.2) and SAS 9.4 (SAS Institute Inc., Cary, NC, USA.) were used for statistical analysis. A two-tailed *P* value < 0.05 was considered statistically significant.

## Result

### Baseline characteristics of study participants

During 4-year follow-up, 1278 CircS and 950 symptomatic knee arthritis new cases were observed with the incidence of 23.1% and 9.7%, respectively. A total of 5525 non-CircS individuals were included in the symptomatic knee arthritis group study, with 350 (6.3%) symptomatic knee arthritis individuals at baseline. Participants with the symptomatic knee arthritis were more likely to be female (56.9% vs. 45.0%), lower educated (81.7% vs. 66.7%), residing in rural areas (81.1% vs. 68.3%), and had higher proportion of MVPA (89.5% vs. 78.9%), stroke (2.9% vs. 1.2%) and heart problem (14.3% vs. 7.4%). A total of 9773 individuals without symptomatic knee arthritis were included in CircS group study at baseline, with 3441 (35.2%) CircS individuals. Compared with the non-CircS group, the CircS group was more likely to be older (59.6 vs. 58.0), female (63.5% vs. 44.5%), residing in urban (40.5% vs. 33.5%), having a higher BMI (49.2% vs. 20.6%), annual household income (22.4% vs. 20.0%), and proportion of stroke (3.4% vs. 1.4%), cancer (1.2% vs. 0.4%), heart problems (16.4% vs. 7.3%) (Table 1). We also compared the characteristics of the people included in the study and those lost to follow-up (Table S1 supplementary material). Individuals lost to follow-up were more likely to be older, male, and have a higher proportion of MVPA, as well as cancer, stroke, and heart problems, compared to those included.

**Table 1:** Characteristics of participants at baseline according to different exposures

Characteristics	Symptomatic knee arthritis (2011)				CircS (2011)		
	No (*N* = 5175)	Yes (*N* = 350)	*P*		No (*N* = 6332)	Yes (*N* = 3441)	*P*
Age, mean (SD)	57.9(8.7)	58.9(8.1)	0.052		58.0(9.1)	59.6(9.0)	< 0.001
Male, n (%)	2848(55.0)	151(43.1)	< 0.001		3516(55.5)	1257(36.5)	< 0.001
Elementary school and below, n (%)	3450(66.7)	286(81.7)	< 0.001		4171(65.9)	2464(71.6)	< 0.001
Married/partnered, n (%)	4668(90.2)	315(90.0)	0.902		5680(89.7)	2956(85.9)	< 0.001
Rural living, n (%)	3534(68.3)	284(81.1)	< 0.001		4209(66.5)	2048(59.5)	< 0.001
BMI (kg/m^2^), n (%)			0.386				< 0.001
< 18.5	420(8.2)	39(11.2)			524(8.3)	84(2.5)	
18.5–24.9	3656(71.1)	236(68.0)			4469(71.1)	1645(48.3)	
25.0–30.0	915(17.8)	59(17.0)			1118(17.8)	1382(40.6)	
≥ 30.0	149(2.9)	13(3.8)			178(2.8)	292(8.6)	
Annual household income (CNY), n (%)			0.424				0.028
≤ 2000	2624(50.7)	178(50.9)			3267(51.6)	1710(49.7)	
2001–10,000	809(15.6)	67(19.1)			953(15.0)	534(15.5)	
10,001–25,000	718(13.9)	48(13.7)			847(13.4)	426(12.4)	
> 25,000	1024(19.8)	57(16.3)			1265(20.0)	771(22.4)	
Drinking, n (%)	2197(42.5)	135(38.6)	0.152		2714(42.9)	1150(33.4)	< 0.001
Smoking, n (%)			0.128				< 0.001
Non-smoker	2842(54.9)	206(58.8)			3472(54.8)	2348(68.2)	
Ex-smoker	439(8.5)	30(8.6)			528(8.4)	292(8.5)	
Current smoker	1893(36.6)	114(32.6)			2331(36.8)	801(23.3)	
MVPA, n (%)	1608(78.9)	128(89.5)	0.002		1914(78.0)	890(68.4)	< 0.001
Cancer, n (%)	19(0.4)	3(0.9)	0.158		28(0.4)	42(1.2)	< 0.001
Stroke, n (%)	63(1.2)	10(2.9)	0.010		87(1.4)	116(3.4)	< 0.001
Heart problem, n (%)	380(7.4)	50(14.3)	< 0.001		461(7.3)	562(16.4)	< 0.001

*CircS* circadian syndrome, *BMI* body mass index, *MVPA* moderate to vigorous physical activity

### Longitudinal association of symptomatic knee arthritis and CircS

Individuals with symptomatic knee arthritis exhibited a significantly higher risk of developing incident CircS, compared to those without symptomatic knee arthritis at baseline (OR = 1.58, 95% *CI*: 1.23–2.02) after adjusted for potential confounders. Participants with CircS had a significantly increased risk of symptomatic knee arthritis compared to those without CircS (OR = 1.49, 95% *CI*: 1.29–1.73) after adjusted for potential confounders (Table 2). Multivariate analysis for the CircS components revealed that short sleep duration (OR = 1.44, 95% *CI*: 1.25–1.67) and depression (OR = 2.24, 95% *CI*: 1.94–2.59) were independently significantly associated with development of symptomatic knee arthritis, while no significantly independent relationships existed between other CircS components and symptomatic knee arthritis. Notably, depression (standardized coefficient *β* = 0.211) had a larger contribution to the knee arthritis development than any other components of CircS, as compared using the standardized regression coefficient.

**Table 2:** Longitudinal association between symptomatic knee arthritis and CircS

Model	Symptomatic knee arthritis (*N* = 5525)			CircS (*N* = 9773)	
	OR (95% CI)	*P*		OR (95% CI)	*P*
Unadjusted	1.74 (1.37,2.18)	< 0.001		1.67 (1.46,1.91)	< 0.001
Model1	1.58 (1.25,2.00)	< 0.001		1.46 (1.27,1.68)	< 0.001
Model2	1.58 (1.23,2.02)	< 0.001		1.49 (1.29,1.73)	< 0.001

Data are presented as odds ratios (95% confidence interval). Model 1 was adjusted for sex and age; Model 2 was additionally adjusted for education, marital status, residence, BMI, annual household income, smoking, drinking, activity, cancer, stroke and heart problem. *CircS* circadian syndrome, *OR* odds ratio; 95% *CI*, 95% confidence interval

**Fig. 2:**
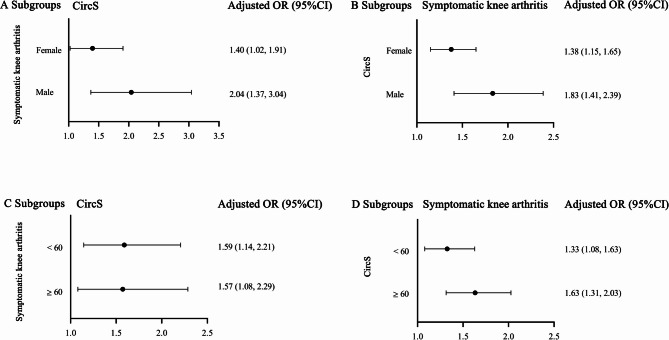
Subgroup analyses of the association between symptomatic knee arthritis and CircS. All models were adjusted for education, marital status, residence, BMI, annual household income, smoking, drinking, activity, cancer, lung disease and heart problem; **A**, the association between knee arthritis and incident CircS stratified by sex; **B**, the association between CircS and incident knee arthritis stratified by sex; **C**, the association between knee arthritis and incident CircS stratified by age; **D**, the association between CircS and incident knee arthritis stratified by age. Notes: CircS, circadian syndrome; OR, odds ratio; 95% *CI*, 95% confidence interval

Subgroup analyses based on age and sex were performed for both the analysis groups (Fig. 2). Both age (symptomatic knee arthritis group: *P*_interaction_=0.297, CircS group: *P*_interaction_ =0.545) and sex (symptomatic knee arthritis group: *P*_interaction_ =0.933, CircS group: *P*_interaction_ =0.504) showed no statistically significant interaction effect on the longitudinal association between symptomatic knee arthritis and CircS. In all subgroups, participants with symptomatic knee arthritis had 39.7%−104.2% increased risk of developing incident CircS, and participants with CircS had 32.5%−83.3% elevated risk of developing incident symptomatic knee arthritis.

### Bidirectional relationship between symptomatic knee arthritis and CircS

According to the data presented in Table 3, The path coefficient (*β*_*1*_) between symptomatic knee arthritis at baseline and CircS at follow-up was estimated to be 0.047 (95% *CI*: 0.028–0.066, *P* < 0.001) and the path coefficient from CircS to symptomatic knee arthritis was 0.066 (95% *CI*: 0.046–0.086, *P* < 0.001). The goodness-of-fit indices indicated a moderate model fit for the cross-lagged model, with a CFI value of 0.868 and a RMSEA value of 0.064. The association remained consistent after grouping by sex and we found that there was no statistically significant difference between the path coefficients *β*_*1*_ and *β*_*2*_ in all models. (Figure S2 supplementary material).

**Table 3:** Cross-lagged model path coefficients between symptomatic knee arthritis and CircS

Path	β	se	*P*	95% CI	RMSEA	CFI
Symptomatic knee arthritis→CircS	0.047	0.010	< 0.001	(0.028,0.066)	0.064	0.868
Symptomatic knee arthritis→Symptomatic knee arthritis	0.321	0.010	< 0.001	(0.301,0.341)		
CircS→Symptomatic knee arthritis	0.066	0.010	< 0.001	(0.046,0.086)		
CircS→CircS	0.439	0.010	< 0.001	(0.420,0.458)		

*CircS* circadian syndrome, 95% *CI*, 95% confidence interval; *RMSEA* Root Mean Square Error of Approximation, *CFI* Comparative Fit Index

### Sensitivity analysis

The results of the binary logistic regression with the raw dataset without imputation indicated the consistent association between the symptomatic knee arthritis and CircS (Table S2 supplementary material).

## Discussion

Using the data of a representative prospective cohort, we found that there was a bidirectional association between symptomatic knee arthritis and CircS. Symptomatic knee arthritis increased the CircS risk and the CircS is also associated with an increased risk of incident symptomatic knee arthritis among middle-aged and older adults in China.

The main finding of this study is that there is a bidirectional association between symptomatic knee arthritis and CircS. An increasing number of previous studies have explored the associations and underlying mechanisms between various metabolic components of CircS and symptomatic knee arthritis. Obesity was positively correlated with increased load on the lower limb joints, which in turn contributes to the elevated risk of developing KOA in overweight individuals [24–26]. The levels of serum tumor necrosis factor-alpha (TNF-*α*), interleukin 1 beta (IL-1*β*) and IL-6 were higher in obese people. The levels of TNF-*α*, IL-1*β* and IL-6 in the synovial fluid, synovial membrane, subchondral bone, and cartilage of OA patients were also found to be relatively high [9]. A positive association between dyslipidemia and KOA has been established [27]. Dysfunctional high-density lipoproteins (HDLs), in conjunction with elevated systemic cholesterol and triglyceride levels, have been linked to the hastened progression of joint pathology and cartilage deterioration in knee osteoarthritis. These metabolic irregularities are known sources of intensified pain in OA, as they lead to synovial activation, abnormal osteogenesis, and heightened occurrences of bone marrow lesions [9]. In a cohort study, Higher blood pressure (both systolic and diastolic) has been observed in individuals with OA compared to those without OA. Increased incidence of OA has been linked to higher systolic blood pressure, which increases the risk of OA in affected patients [28]. A previous study identified OA as a significant risk factor for cardiovascular disease, implying a potential association between OA and vascular damage [29]. Several studies have found a bidirectional association between MetS and osteoarthritis [10]. Pan and colleagues from Australia reported that metabolic syndrome was found to be a significant predictor of pain progression patterns in individuals with knee pain [30]. It is consistent with our study of the association between symptomatic knee arthritis and CircS. Individuals who experience both sleep deprivation and depression have a higher propensity to develop arthritis. In a cohort study, Depression patients had a higher risk of developing arthritis compared to individuals without depression, and the occurrence of arthritis further elevated the risk of incident depression [12]. This study integrates metabolic factors, depression, and sleep disturbances as a whole to investigate the bidirectional associations with symptomatic knee arthritis. Additionally, depression contributed the most to the occurrence of knee arthritis among the Circs components, which may provide new insights into the multifactorial pathology of knee arthritis.

Studies have demonstrated the association between circadian disruption and all components of CircS. Sleep loss may induce a state of chronic inflammation, promote loss of oxidative stress and impair neuroprotection, which can result in more severe circadian and sleep dysfunctions [31]. Dysfunction of the circadian clock has a significant impact on host lipid metabolism and accelerates the development of obesity [16]. Sleep duration and quality have been shown to impact the circadian rhythm of blood pressure [32]. Furthermore, Short sleep duration exerted an adverse causal effect on arthritis and the results remained significant after controlling other factors [13, 33]. Circadian hormones including melatonin, thyroid-stimulating hormone and cortisol had a significant effect on OA by leading to greater expression of pro-inflammatory cytokines and cartilage degenerative enzymes in articular cartilage. The alterations described could prompt cartilage erosion, synovial inflammation, and osteophyte formation, all of which are potential contributors to cartilage degeneration and the progressive advancement of OA [34]. Based on the mechanism by which sleep influenced metabolism and subsequently impacted knee arthritis, future pharmacological research could explore interventions targeting the sleep-metabolism pathway as a potential strategy to prevent the progression of knee arthritis. Sleep deprivation was associated with gut microbiota dysbiosis, which in turn affects the incidence of OA. In mice, sleep deprivation was linked to decreased melatonin levels, which were associated with a decrease in gut microbial diversity and richness as well as colonic mucosal injury. Inflammation caused by sleep deprivation can be mediated by gut microbiome [35]. The composition and detrimental alterations of gut microbiota have been implicated in the pathogenesis of musculoskeletal disorders. Specific microbes, fermentable fibers and bacterial metabolites may prevent knee joint damage and the composition of gut microbiota influenced the metabolic process of arthritis [36, 37].

There are several limitations in this study that need to be acknowledged. The assessment of pain in knee arthritis is limited by the lack of defined thresholds. Although different from the criteria used in other studies, it has been validated and utilized in prior research [7, 21]. Secondly, Due to the data collection approach of the cohort, it is not possible to differentiate between osteoarthritis and rheumatoid arthritis. Thirdly, individuals being excluded from the study sample were more likely to be older, male, residing in urban areas, and had a worse state of health. This may have introduced selection bias, which may have had important impacts on the results. Finally, although this study has accounted for several potential confounders, it is acknowledged that certain confounding factors, such as dietary habits, were not included in the analysis and may have an impact on the results [38].

## Conclusion

To conclude, the findings of this study indicate bidirectional associations between symptomatic knee arthritis and CircS. This highlights the importance of careful and timely monitoring of patients with symptomatic knee arthritis and CircS to prevent their health from getting worse. Given the limited availability of data, additional research should be undertaken to elucidate the underlying mechanisms between knee arthritis and CircS.

## Supplementary Information

Supplementary Material 1

## Data Availability

The data utilized in this study are publicly available for download at the following website: https://charls.pku.edu.cn/.

## Abbreviations

RA: Rheumatoid arthritis
OA: Osteoarthritis
KOA: Knee osteoarthritis
CircS: Circadian syndrome
MetS: Metabolic syndrome
CHARLS: China health and retirement longitudinal study
CES-D -10: 10-item version of the center for epidemiological studies depression scale
MVPA: Moderate to vigorous physical activity
WHO: World health organization
OR: Odds ratio
CI: Confidence interval
RMSEA: Root mean square error of approximation
CFI: Comparative fit index
TNF-α: Factor-alpha
IL-1β: Interleukin 1 beta
HDLs: High-density lipoproteins
