# Supplementary material for: Bidirectional association between symptomatic knee arthritis and circadian syndrome among middle-aged and older population: an analysis from the China health and retirement longitudinal study
Source: BMC Public Health. 2025 Nov 3;25:3753. doi: 10.1186/s12889-025-25100-1 (PMC12581250; doi:10.1186/s12889-025-25100-1)
Supplement: Supplementary file 1 — Supplementary Material 1 [file 12889_2025_25100_MOESM1_ESM.pdf]

Supplementary Materials

Supplemental Table 1. Characteristics of participants included and excluded.

Supplemental Table 2. Longitudinal association between symptomatic knee arthritis and CircS.

Supplemental Figure 1. Simple diagram of cross-lagged path analysis model; 2011 represents baseline and 2015 represents follow-up. Notes, CircS, circadian syndrome.

Supplemental Figure 2. Cross-lagged path with statistical significance; *, P<0.01; 2011 represents baseline and 2015 represents follow-up. Notes, CircS, circadian syndrome.

Supplemental Table 1. Characteristics of participants included and excluded.

Characteristics	Symptomatic knee arthritis (2011)			CircS (2011)		
	Included (N=5525)	Excluded (N=2402)	*P*	Included (N=9773)	Excluded (N=1721)	*P*
Age, mean (SD)	58.0(8.7)	59.6(11.0)	<0.001	58.6(9.1)	61.6(11.1)	<0.001
Male, n (%)	2999(54.3)	1383(57.6)	0.007	4773(48.8)	893(51.9)	0.020
Elementary school and below, n (%)	3736(67.6)	1538(64.0)	0.002	6635(67.9)	1111(64.6)	0.007
Married/partnered, n (%)	4983(90.2)	2066(86.0)	<0.001	8636(88.4)	1414(82.2)	<0.001
Rural living, n (%)	3818(69.1)	1342(55.9)	<0.001	6257(64.0)	827(48.1)	<0.001
BMI (kg/m^2), n (%)			0.006			<0.001
<18.5	459(8.4)	254(10.8)		608(6.3)	163(9.7)	
18.5-24.9	3892(70.9)	1618(68.7)		6114(63.1)	1028(61.3)	
25.0-30.0	974(17.8)	421(17.9)		2500(25.8)	413(24.6)	
⩾30.0	162(2.9)	61(2.6)		470(4.8)	74(4.4)	
Annual household income (CNY), n (%)			<0.001			<0.001
⩽2000	2802(50.7)	1340(55.8)		4977(50.9)	929(54.0)	
2001–10,000	876(15.9)	299(12.5)		1487(15.2)	199(11.5)	
10,001–25,000	766(13.8)	275(11.4)		1273(13.1)	213(12.4)	
>25,000	1081(19.6)	488(20.3)		2036(20.8)	380(22.1)	
Drinking, n (%)	2332(42.2)	1048(43.7)	0.241	3864(39.6)	699(40.6)	0.396
Smoking, n (%)			0.098			<0.001
Non-smoker	3048(55.2)	1290(53.7)		5820(59.6)	964(56.0)	
Ex-smoker	469(8.5)	239(10.0)		820(8.4)	222(12.9)	
Current smoker	2007(36.3)	873(36.3)		3132(32.0)	535(31.1)	
MVPA, n (%)	1736(79.6)	615(71.3)	<0.001	2804(74.7)	369(61.9)	0.001
Cancer, n (%)	22(0.4)	22(0.9)	0.004	70(0.7)	24(1.4)	0.004
Stroke, n (%)	73(1.32)	55(2.3)	0.002	203(2.1)	70(4.1)	<0.001
Heart problem, n (%)	430(7.8)	222(9.3)	0.030	1023(10.5)	245(14.3)	<0.001

Notes: CircS, circadian syndrome; BMI, body mass index; MVPA, moderate to vigorous physical activity.

Supplemental Table 2. Longitudinal association between symptomatic knee arthritis and CircS.

	Symptomatic knee arthritis (N=5525)		CircS (N=9773)	
	OR (95% *CI*)	*P* value	OR (95% *CI*)	*P* value
Unadjusted	1.74 (1.37,2.18)	<0.001	1.67 (1.46,1.91)	<0.001
Model1	1.58 (1.25,2.00)	<0.001	1.46 (1.27,1.68)	<0.001
Model2	2.07 (1.41,3.05)	<0.001	1.45 (1.14,1.84)	0.002

Data are presented as relative risks (95% confidence interval). Model 1 was adjusted for sex and age; Model 2 was adjusted for sex, age, education, marital status, residence, BMI, annual household income, smoking, drinking, activity, cancer, lung disease and heart problem. Notes, CircS, circadian syndrome; OR, odds ratio; 95% *CI*, 95% confidence interval.

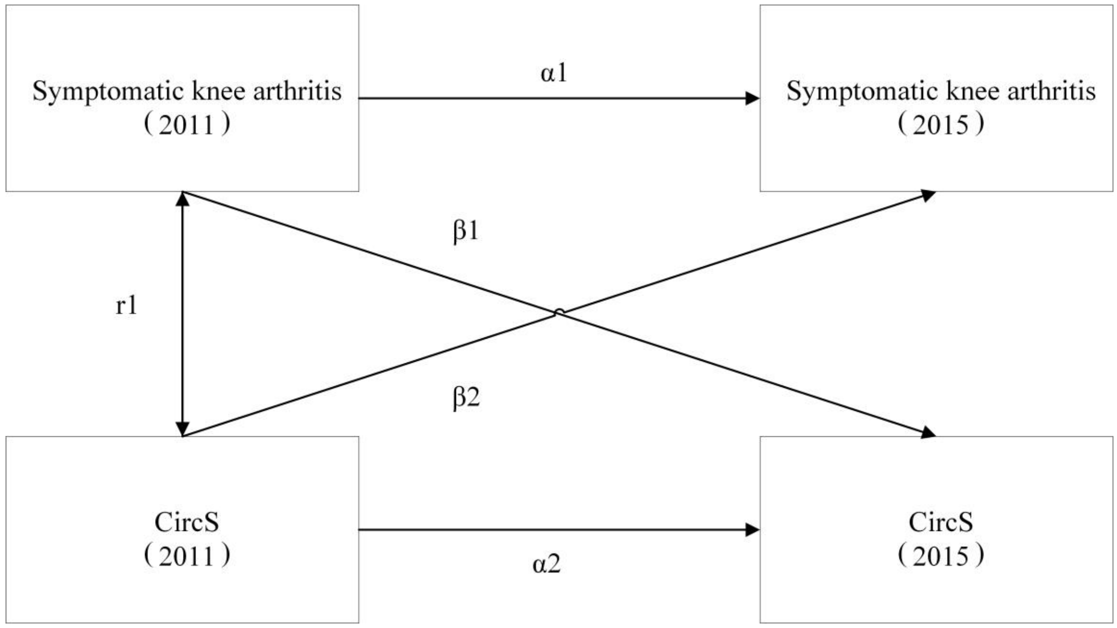

Supplemental Figure 1. Simple diagram of cross-lagged path analysis model; 2011 represents baseline and 2015 represents follow-up. Notes, CircS, circadian syndrome.

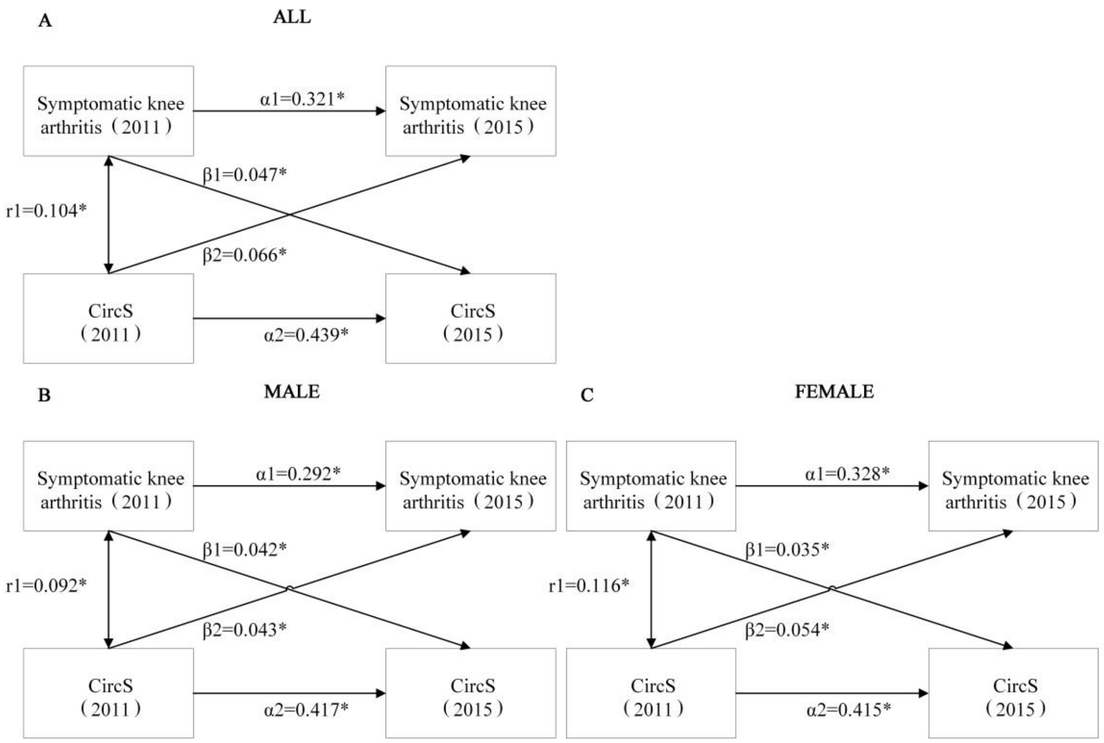

Supplemental Figure 2. Cross-lagged path with statistical significance; *, P<0.01; 2011 represents baseline and 2015 represents follow-up. Notes, CircS, circadian syndrome.
